# Supplementary material for: A Review of the Effect of Formic Acid and Its Salts on the Gastrointestinal Microbiota and Performance of Pigs
Source: Animals (Basel). 2020 May 19;10(5):887. doi: 10.3390/ani10050887 (PMC7278376; doi:10.3390/ani10050887)
Supplement: Supplementary file 1 [file animals-10-00887-s001.pdf]

Title: A review of the effect of formic acid and its salts on the gastrointestinal microbiota and performance of pigs.

Diana Luise [1], Federico Correa [1], Paolo Bosi [1], Paolo Trevisi [1]

[1] Department of Agricultural and Food Sciences (DISTAL), University of Bologna, 40127 Bologna, Italy; diana.luise2@unibo.it (D.L.) ; federico.correa2@unibo.it (F.C.) ; paolo.bosi@unibo.it (P.B.) ; paolo.trevisi@unibo.it (P.T.)

* Correspondence: diana.luise2@unibo.it (D.L)

Supplementary Table 1. Summary the effects of organic acid on the pig microbiota.

Acidifier	Quantity	Phase	Intestinal tract	Alpha indices	Beta-diversity	Difference in taxa	Direction	Taxonomy level	Reference
Formic acid	1.4g/kg or 6.4 g/kg	weaning	Jejunum	↓ Chao1 (6.4g/kg)	=	Gemella	↓ (1.4g/kg/ 6.4g/kg)	Genus	[8]
						Lactobacillus	↓ (1.4g/kg/ 6.4g/kg)	Genus	
						Parvimonas	↓ (1.4g/kg/ 6.4g/kg)	Genus	
						Streptococcus	↓ (1.4g/kg) ↑ (6.4 g/kg)	Genus	
						Turicibacter	↓ (1.4g/kg)	Genus	
						Acinetobacter	↓ (6.4g/kg)	Genus	
						Fusobacterium	↓ (6.4g/kg)	Genus	
						Leuconostoc	↓ (6.4g/kg)	Genus	
Formic acid	0.60%	weaning	Ileum	=	=	-	-	-	[84]
Benzoic acid	2 g/kg	weaning	Colon	=	≠	Bacteroides	↑	Genus	[58]
						Prevotella	↓	Genus	
						Unclassified S24-7	↑	Genus	
Sodium butyrate	0.20%	growing	cecum	↓ Chao1	/	Firmicutes	↓	Phylum	[59]
						Bacteriodetes	↑	Phylum	
						Proteobacteria	↓	Phylum	
						Tenericutes	↓	Phylum	
						Synergistetes	↓	Phylum	

						Flavobacterium	↑	Genus	
						Dorea	↓	Genus	
						Blautia	↓	Genus	
						Desulfovibrio	↓	Genus	
						Coprococcus	↓	Genus	
						Succinivibrio	↓	Genus	
						[Ruminococcus]	↓	Genus	
Sodium butyrate (encapsulatded)	1g/kg + antibiotic	weaning	Colon	↑ Shannon	/	Bacteroidetes	↑	Phylum	[10]
				↓ Simpson		Lactobacillaceae	↓	Family	
						Clostridiaceae	↑	Family	
						Ruminococcaceae	↑	Family	
						Lachnospiraceae	↑	Family	
			Ileum	=	/	Lactobacillaceae	↓	Family	
						Clostridiaceae	↑	Family	
						Pasteurellaceae	↓	Family	
						Enterobacteriaceae	↓	Family	
